# Individual IOL Surface Topography Analysis by the WaveMaster Reflex UV

**DOI:** 10.1155/2013/363742

**Published:** 2013-05-28

**Authors:** Marc Kannengießer, Achim Langenbucher, Edgar Janunts

**Affiliations:** Department of Experimental Ophthalmology, Saarland University, Kirrberger Straße 100, 66424 Homburg, Germany

## Abstract

*Purpose*. In order to establish inspection routines for individual intraocular lenses (IOLs), their surfaces have to be measured separately. Currently available measurement devices lack this functionality. The purpose of this study is to evaluate a new topography measurement device based on wavefront analysis for measuring individual regular and freeform IOL surfaces, the “WaveMaster Reflex UV” (Trioptics, Wedel, Germany). *Methods*. Measurements were performed on IOLs with increasingly complex surface geometries: spherical surfaces, surfaces modelled by higher-order Zernike terms, and freeform surfaces from biometrical patient data. Two independent parameters were measured: the sample's radius of curvature (ROC) and its residual (difference of sample topography and its best-fit sphere). We used a quantitative analysis method by calculating the residuals' root-mean-square (RMS) and peak-to-Valley (P2V) values. *Results*. The sample's best-fit ROC differences increased with the sample's complexity. The sample's differences of RMS values were 80 nm for spherical surfaces, 97 nm for higher-order samples, and 21 nm for freeform surfaces. Graphical representations of both measurement and design topographies were recorded and compared. *Conclusion*. The measurements of spherical surfaces expectedly resulted in better values than those of freeform surfaces. Overall, the wavefront analysing method proves to be an effective method for evaluating individual IOL surfaces.

## 1. Introduction

Cataract surgery has been the most frequent surgical procedure for the last decades. Its primary goal was to restore the patient's vision. Currently, the patient's satisfaction and the restoration of the target refraction are of heightened interest. This leads to extensive research on more sophisticated lens surfaces which can eliminate even higher-order aberrations of the patient's optical system. With the aim of maximal improvement of the patient's visual performance, the latest developments focus on IOLs with customized freeform surface geometries which compensate corneal aberrations [1–3]. They are produced by a nonpolishing lathing process which offers new challenges for postprocessing lens quality inspection.

To our knowledge, there is no system available for scanning individual IOL surfaces. Currently, the quality inspection of conventional IOLs is done directly in the production chain by interferometry, deflectometry, or wavefront sensors. Interferometry devices compare signals from the sample's surface and focus mainly on spherical surfaces. They are considered the golden standard for optical lens surface inspection. Their limited dynamic range, however, limits their application to basic lens geometries, such as rotationally symmetric or aspheric IOL surfaces. More complex surfaces require sophisticated measures to stay within their limited dynamic range [4, 5]. Deflectometric devices evaluate an IOL surface by projecting a pattern (e.g., lines, chessboard patterns) and analyse its deformation in a camera image [5, 6]. Most setups work in reflection mode with wavelengths in the visible range. Therefore, light is partly reflected off the front surface, while a major part of light is transmitted and reflected off the back side. Both signals are simultaneously received at the detector and interfere with each other. This leads to artefacts and disturbs the measurement. As the IOL is supposed to be implanted after having passed inspection, exposing it to fluids or wax to suppress the back surface reflections is unacceptable. Topography measurements by wavefront sensors are affected by the same issues because current wavefront sensors operate in the visible spectrum.

In order to measure individual surfaces under sterile conditions, we introduce a new device for measuring individual IOL surface topographies: The WaveMaster Reflex UV (Trioptics, Wedel, Germany). Like its predecessors, the WaveMaster IOL and the WaveMaster Pro Reflex, it operates by a Shack-Hartmann sensor (SHS) setup [7]. Both predecessors are, however, not designed for measuring the IOL topography but rather its visual performance. They operate with radiation in the visible range. Therefore, signals from the IOL back side are always overlapped with front surface signals. One can, though, suppress those signals by blackening the back side of the IOL, but this would desterilize the IOL. The novelty of the WaveMaster Reflex UV is its operation in th e near-UV range of 365 nm. The radiation in this wavelength is greatly absorbed inside the IOL material, so that undesired back reflexes are suppressed. The novel achievement of using near-UV radiation in an SHS-based IOL topography scanner enables the sterile measurement of individual IOL surface topographies.

The aim of this paper is to demonstrate the applicability of the new WaveMaster Reflex UV in the field of measuring individual IOL surface topographies.

## 2. Materials and Methods

The measurements were performed in a lab environment at a temperature of 22°C. 

All measured IOLs are made from Contamac blanks [8]. We perform measurements on three kinds of surface topographies The first group contains spherical surface geometries with radii of curvature (ROCs) between 6 mm and 20 mm. 

The second group consists of six samples with surface geometries representing a superposition of higher-order aberrations: Zernike coefficients of coma, astigmatism, trefoil, and tetrafoil are added to a spherical surface with a base ROC of 11,5 mm to model higher-order aberrations [15]:

(1) Z(x,y)c=ZSphere(x,y)+ZComa(x,y)     +ZTrifoil(x,y)+ZTetrafoil(x,y)ZSphere(x,y)=11,52−(x2−y2),ZComa(x,y)=K·((−2)·mx+3·mx·(mx2+my2)),ZTrifoil(x,y)=K·(x3−3·x·y2),ZTetrafoil(x,y)=−K·(x4−6·x2y2+y4),mx=x·cos⁡(Φ)−y·sin(Φ),my=x·sin(Φ)−y·cos⁡(Φ).

The six surfaces are calculated according to the following set of parameters:

 Sample 1: Φ = 0°; *K* = 0.0001;
 Sample 2: Φ = 0°; *K* = 0.0003;
 Sample 3: Φ = 0°; *K* = 0.0005;
 Sample 4: Φ = 180°; *K* = 0.0001;
 Sample 5: Φ = 180°; *K* = 0.0002;
 Sample 6: Φ = 180°; *K* = 0.0003.

They are referred to as “higher-order samples.”

The third group holds two customized lenses derived from biometric patient data [1–3]. A general quadric surface is described by the following equation:

(2) S(x,y,z)=Ax2+By2+Cz2+2Dxy+2Eyz     +2Fxz+2Gx+2Hy+2Iz+K=0.

Alternatively, the surface can be described in matrix nomenclature by

(3) $$\begin{array}{c} S=\left[\begin{array}{cccc} A & D & F & G\\ D & B & E & H\\ F & E & C & I\\ G & H & I & K \end{array}\right]. \end{array}$$

The coefficients of the two surfaces under investigation are

Sample 1

(4) $$\begin{array}{c} \left(\begin{array}{cccc} 1 & -0.094 & -0.065 & 0.025\\ -0.094 & 1.135 & 0.101 & -0.039\\ -0.065 & 0.101 & -2.897 & 7.660\\ 0.025 & -0.039 & 7.660 & -5.211 \end{array}\right). \end{array}$$

Sample 2

(5) $$\begin{array}{c} \left(\begin{array}{cccc} 1 & -0.028 & -0.131 & 0.060\\ -0.028 & 0.992 & -0.272 & 0.007\\ -0.131 & -0.272 & -7.195 & 11.259\\ 0.060 & 0.007 & 11.259 & -4.541 \end{array}\right). \end{array}$$

This group is labelled “freeform samples.”

**Figure 1:**
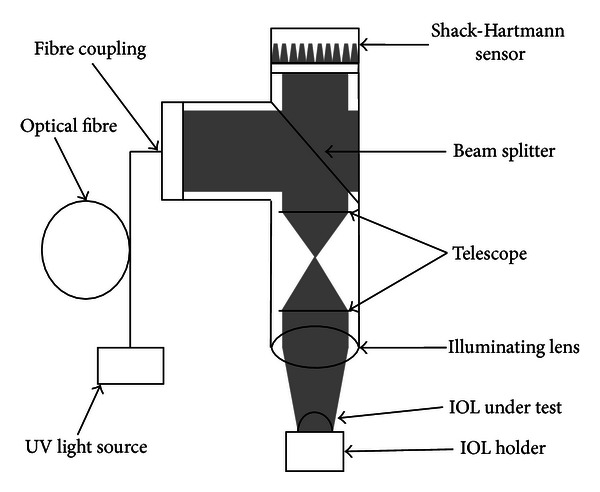
Scheme of the beam path of the WaveMaster Reflex UV. The light is coupled into the left. It hits the beam splitter and is then imaged to the sample. The reflected part passes back through the beam splitter. It is imaged onto the SHS which detects the deviation from a purely spherical wavefront.

The WaveMaster Reflex UV is an SHS-based IOL topography scanner in reflection mode and measures the wavefront aberrations caused by the IOL front surface (Figure 1). The fibre-coupled UV-radiation (365 nm) is directed to the sample by a beam-splitter. The main part of the incoming beam is reflected by the first surface, while the rest is absorbed in the lens because of its high absorption in the near-UV range. The reflected part passes through the beam splitter and is imaged by a microlens array to the SHS. The components are optimized for UV radiation and measure the deviation from a spherical wavefront. This deviation is labelled “residual” in this work.

**Figure 2:**
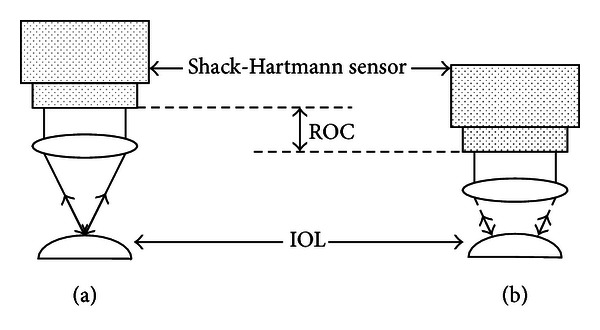
Scheme of the WaveMaster Reflex UV's measurement positions. (a) Shows the CE position of the sample, (b) sketches the MP. The path difference along the optical axis corresponds to the sample's best-fit ROC.

**Figure 3:**
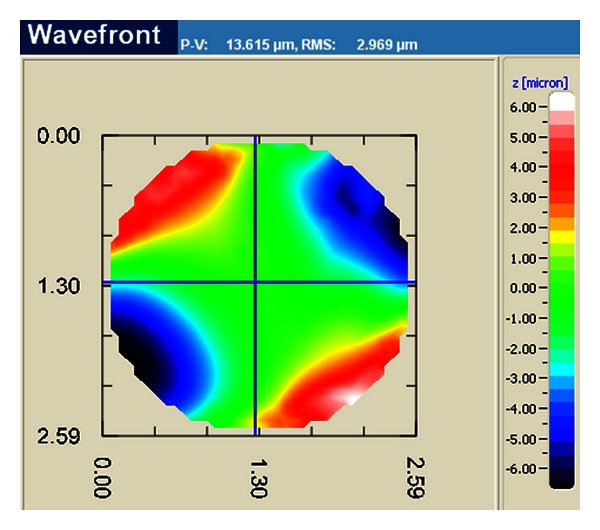
Screenshot of a measured residual of a toric sample. The residual shows a saddle-shaped pattern. An area of roughly 2.6 mm diameter was imaged. The device automatically calculates the RMS and P2V values on the top bar.

Figure 2 shows a standard measurement procedure: every sample is measured along the optical axis in two critical positions: the cat's eye position (CE) and measurement position (MP). They are similar to interferometric measurements. In the cat's eye position, the surface's apex is imaged; each ray is reflected in its opposite direction towards the sensor. The measurement position is distinguished by the fact that each ray is reflected back to its original position (see arrows in Figure 2(b)); the entire area is imaged. In this position, the device measures the residual (Difference of sample topography and its best-fit sphere for the measured area) and displays it in a colour-coded map (Figure 3). The user moves the sensor setup by a motorized stage along the optical axis to find the CE and MP positions. Their axial difference is the sample's best-fit ROC. This is the common measurement procedure known from interferometric setups.

The device features the fitting of the measured residual to a Zernike composition, displaying the values of the respective Zernike coefficients [9, 10].

The defocus term is used by the device as the criterion for acquiring the CE and MP positions along the optical axis. In those positions, it is required to be below lambda/10, that is, 37 nm.

**Figure 4:**
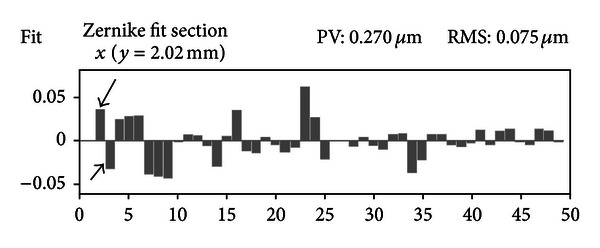
Zernike coefficient decomposition: The software fits a Zernike function to the measured topography. It enables a coefficient analysis of the resulting Zernike fit. The values for each coefficient are listed as numbers or, in case of the above image, displayed by a bar graph. The coefficients for tilt in *x* and *y* directions are marked by arrows. They guide the operator in centering the sample. Measuring a decentered sample will lead to higher tilt values. By lateral adjustment of the sample holder, the operators can find the position where they are minimal. The measured IOL is then centered.

The coefficients for tilt in the *x* and *y* direction are labelled with arrows in Figure 4. They guide the user in the sample centration process. The sample is fixed and aligned to its holder during the production process. The holders themselves are inserted to the mount of the device's moving table. A tilt of the lens itself is unexpected. Therefore, any measured tilt is associated with a lateral misalignment of the sample with respect to the device's optical axis. A laterally centered stage results in minimal tilt coefficients.

**Figure 5:**
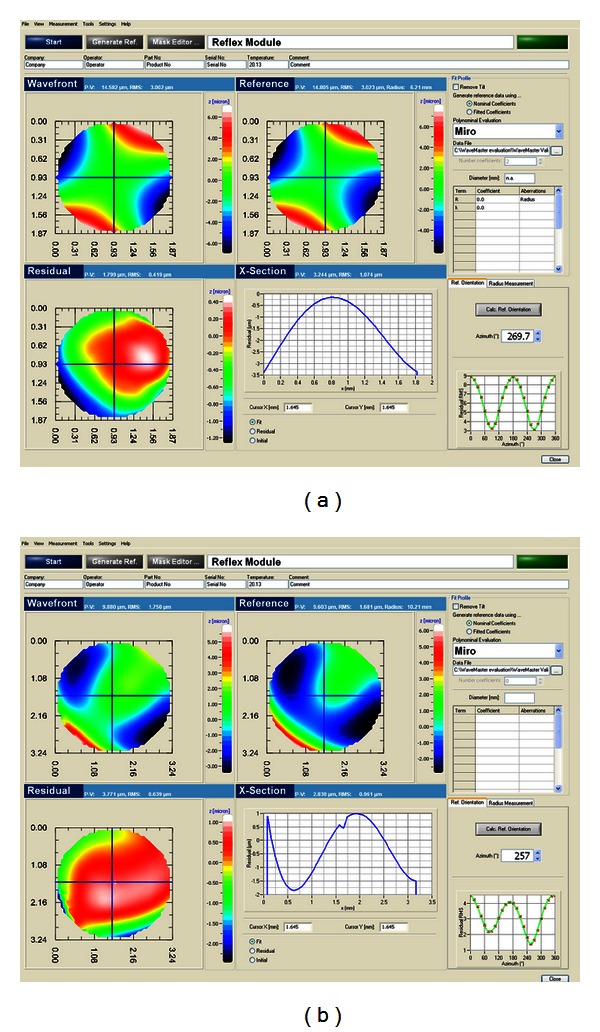
Residual comparison of the freeform surfaces. The upper half lists the results for the first surface, the lower half shows the comparison of the second freeform surface.

The measured residual can be compared against a set of surface geometries such as spherical and aspherical, toric and user-defined freeform surfaces. The design topography is limited to the measured part of the surface to ensure comparability. The analysis screen consists of four panels (Figure 5): The first three (upper left, upper right, and lower left, titled “Wavefront,” “Reference,” and “Residual”, resp.) hold colour-coded plots. The “Wavefront” and “Reference” panels show the measured residual and the design residual with a best-fit sphere subtracted from its topography. The corresponding ROC is shown to the right, next to the label “Reference.” The “Residual” panel shows the difference between the measured residual in “Wavefront” and the design residual in “Reference.” The RMS and P2V values are calculated for all three panels and listed to the right of the labels. 

In the evaluation process, we record the following parameters: the sample's best-fit ROC and its residual. The ROC serves as representation of its lower-order aberrations [11]. It has a major effect on the IOL refractive power and is therefore primarily associated with its basic visual performance. The theoretical value for the ROC is either given by the manufacturer in case of spherical surfaces or derived from the design data by the analysis software of the WaveMaster Reflex UV. 

The residual evaluation consists of two parameters: the RMS and P2V values [12–14]: 

Let *z*_*i*_(*x*_*i*_, *y*_*i*_) be the height value for the *i*th pixel from the total of *n* pixels; then

(6) $$\begin{array}{c} \text{RMS}=\frac{\sqrt{\sum_{i}^{}{\left(z_{i}\left(x_{i},y_{i}\right)-\tilde{z}\right)}^{2}}}{n},\;\tilde{z}=\sum_{i}z_{i}\left(x_{i},y_{i}\right),\\ \text{P}2\text{V}=\max\left(z_{i}\left(x_{i},y_{i}\right)\right)-\min\left(z_{i}\left(x_{i},y_{i}\right)\right). \end{array}$$

The RMS and P2V values of spherical lenses are supposed to be zero. In case of nonspherical lenses, the measurement values have to match the corresponding values of the design data. As the RMS and P2V values represent averaged values of a measured residual, they serve to discern any major deviations between design and actual measurements. They do not reveal any information about the location of defects. This point is addressed by comparing the measured residual map against the design residual (see Figure 5).

## 3. Results

### 3.1. ROC Measurements

Table 1 contains the ROC measurements of spherical surfaces. The ROC ranges from 6 mm to 20 mm with a step size of 2 mm. Between 12 mm and 16 mm, a smaller step size of 0.5 mm was chosen. The measured and the design ROCs are listed in the second column. The fourth column holds the difference between measurement and design in *μ*m. The average value for the ROC differences between design and measurement was calculated to 18 *μ*m with a standard deviation (SDV) of 12 *μ*m. 

The ROC measurements for the higher-order surface geometries are listed in Table 2. The average ROC difference between measurement and design was calculated to be 36 *μ*m with a standard deviation of 5 *μ*m.

Table 3 holds ROC measurements for two IOLs with freeform geometries. As only two surfaces are measured here, the data is provided for descriptive purposes only.

The values for the measured residual of the spherical surfaces are listed in Table 4. Their average values are calculated to be 79 nm/422 nm with a standard deviation of 49 nm/149 nm. 

Table 5 shows the results for the residual analysis of the higher-order samples. The last two columns hold the differences between measurement and design for the respective values of RMS and P2V. The average values for the differences are found in the last two lines and are calculated to be 97 nm/415 nm with corresponding values for their standard deviations of 99 nm/439 nm. 

The results for the residual analysis are found in Table 6. The resulting differences between measurement and the design data are in the range previously measured: Several 10 nm for RMS values and several 100 nm for the P2V values. Although the two surface geometries are quite different from each other, the numbers are in the same order of magnitude.

### 3.2. Residual Maps of Patient Data

The results for freeform surface 1 are shown in Figure 5(a). The surface shows a saddle-shaped pattern, typical for toric surfaces. A direct comparison of “Wavefront” with “Reference” may lead to the conclusion that the measured residual matches the design residual. However, looking at the “Residual” panel reveals the major differences in the peripheral areas to the left and to the bottom of the display. 

The graphical residual analysis for the second freeform surface is listed in Figure 5(b). The major differences between measurement and design data are located in the centre and calculated to be about 1 *μ*m. The cross-section panel reveals a slight dip in the centre with a depth of about 0.2 *μ*m.

## 4. Discussion

The measurement of individual IOL surfaces by the wavefront analyser in the near-UV range is straightforward and accurate. Measurements on clinically available topographers revealed that there can be only a limited range of ROCs measureable by the device [15]. In contrast, the device is able to measure all ROCs between 6 mm and 20 mm. The average deviations are less than 20 *μ*m for spherical samples. This might be due to the *z*-stage's moving precision or manufactoring precision of the sample. The small standard deviation in the ROC acquisition demonstrates the minor amount of statistical errors in the acquisition of the CE and MP positions. The measurements on higher-order samples result in larger ROC differences between measurement and design data. The average value is with 36 *μ*m higher than in case of spherical surfaces. As individual freeform components of varying magnitudes are introduced, the concept of a definitive ROC is ambiguous. The sample's design ROC is calculated according to the least-mean-squares method while the measured ROC is acquired as the axial difference between CE and MP positions. The standard deviation of 5 *μ*m indicates that the ROCs of higher-order surface geometries are accurately measured. In case of the freeform IOL surfaces, the deviations are much higher compared to the previous ones. They consist of values around 340 *μ*m. This increase is caused by the increasingly complex surfaces. Calculating a ROC is an approximation to the measured area, which explains the larger deviations. 

Measuring the RMS and P2V values of spherical surfaces shows the precision of the device. The values are well below 1 *μ*m, with the RMS values being one order of magnitude less than their corresponding P2V values. Since the design data consist of spherical surface geometries, those numbers directly reflect the manufacturing and measurement precision. The measurements on the higher-order samples match their respective design data. From Ho 1 to Ho 3 there is an increase of values, as is the case with Ho 4 through Ho 6. Ho 3 has the highest amount of values for both RMS and P2V values, which is in accordance with it having the highest amplitudes of Zernike coefficients. Ho 2 and Ho 6 share the same amplitude in their Zernike terms (0.003), with different angle values (0° and 180°). Hence, it was expected that the measurements of those samples should give equal values. This is experimentally confirmed. The surface geomtries are more complex; therefore, the standard deviation is higher than in the case of measuring spherical samples. The measurements of the freeform samples lead to similar results. 

The measurements of individual freeform surfaces result in smaller values for the RMS and P2V differences than in the case for higher-order sample geometries.

Looking at the graphical analysis of the first freeform surface, the patterns for measurement and its design data closely match. The most critical deviations are located in the lower left part of the measurement area. We conclude that there is a slight decentration of the sample which results in the pattern seen in Figure 5. This issue can be solved by an individual postprocessed centration of the IOL. The patterns of the second freeform surface differ more. Particularly the blue ridge in the lower left part of the “Reference” panel is not seen in the wavefront graph. We attribute the reason for this disaccordance to a decentration of the sample. The measurements on the residual maps lead to the conclusion that the graphical evaluation is an accurate method to describe differences in topography. 

Although convex and concave lens surfaces can be measured by the WaveMaster Reflex UV, we limit this study to the measurement of biconvex IOL designs.

## 5. Conclusion

The WaveMaster Reflex UV represents a major advancement in the application of measuring individual IOL surfaces, which was impossible if the inspected IOL was required sterile for implantation. The operation in the near-UV range ensures the suppression of reflexes from the IOL back side without desterilizing the IOL. The device operates on a wide range of ROCs with smooth and nonpolished surfaces. The software's capability of measuring and analysing in real time makes it applicable for quality testing in the field of freeform IOL production and manufacturing. Future measurements will show the limits of the device's range in applicability.

## Figures and Tables

**Table 1:** ROC measurement of spherical surfaces.

Sample	Measured ROC/mm	Design ROC/mm	ROC Difference/*µ*m
Sph 6	6.013	6.000	13
Sph 10	10.020	10.000	20
Sph 12	12.012	12.000	12
Sph 12.5	12.534	12.500	34
Sph 13	13.029	13.000	29
Sph 13.5	13.511	13.500	11
Sph 14.5	14.522	14.500	22
Sph 15	15.006	15.000	6
Sph 15.5	15.502	15.500	2
Sph 16	16.022	16.000	22
Sph 18	18.000	18.000	0
Sph 20	20.041	20.000	41

		Average	18
		SDV	12

**Table 2:** ROC measurements for higher-order samples.

Sample	Measured ROC/mm	Design ROC/mm	ROC Difference/*µ*m
Ho 1	11.475	11.500	25
Ho 2	11.461	11.500	39
Ho 3	11.464	11.500	36
Ho 4	11.461	11.500	39
Ho 5	11.462	11.500	38
Ho 6	11.461	11.500	39

		Average	36
		SDV	5

**Table 3:** ROC measurements for freeform surface geometries.

Sample	Measured ROC/mm	Design ROC/mm	ROC Difference/*µ*m
Freeform 1	6.551	6.210	341
Freeform 2	10.541	10.210	331

**Table 4:** Residual analysis for spherical surfaces.

Sample	Measured RMS/*µ*m	Measured P2V/*µ*m
Sph 6	0.060	0.319
Sph 10	0.051	0.308
Sph 12	0.066	0.369
Sph 12.5	0.061	0.400
Sph 13	0.068	0.435
Sph 13.5	0.044	0.305
Sph 14.5	0.060	0.344
Sph 15	0.233	0.844
Sph 15.5	0.066	0.505
Sph 16	0.046	0.325
Sph 18	0.098	0.564
Sph 20	0.100	0.346

Average	0.079	0.422
SDV	0.049	0.149

**Table 5:** Residual analysis for higher-order surfaces.

Sample	Measurement		Design		Difference	
	RMS/*µ*m	P2V/*µ*m	RMS/*µ*m	P2V/*µ*m	RMS/nm	P2V/nm
Ho 1	0.114	0.533	0.034	0.263	80	270
Ho 2	0.251	1.353	0.143	1.125	108	228
Ho 3	0.333	2.279	0.255	1.938	78	341
Ho 4	0.166	0.883	0.046	0.306	120	577
Ho 5	0.151	0.943	0.096	0.641	55	302
Ho 6	0.263	1.617	0.124	0.845	139	772

				Average	0.097	0.415
				SDV	0.099	0.439

**Table 6:** Residual analysis for freeform surface geometries.

Sample	Measurement		Design		Difference	
	RMS/*µ*m	P2V/*µ*m	RMS/*µ*m	P2V/*µ*m	RMS/nm	P2V/nm
Freeform 1	3.002	14.582	3.023	14.805	21	223
Freeform 2	1.750	9.880	1.681	9.603	69	277
